# Study of the Interplay Among Melt Morphology, Rheology and 3D Printability of Poly(Lactic Acid)/Poly(3-Hydroxybutyrate-Co-3-Hydroxyvalerate) Blends

**DOI:** 10.3390/jfb16010009

**Published:** 2025-01-01

**Authors:** Marco Costantini, Flavio Cognini, Roberta Angelini, Sara Alfano, Marianna Villano, Andrea Martinelli, David Bolzonella, Marco Rossi, Andrea Barbetta

**Affiliations:** 1 Institute of Physical Chemistry, Polish Academy of Sciences, 01-224 Warsaw, Poland; 2 Department of Basic and Applied Sciences for Engineering, Sapienza University of Rome, 00161 Rome, Italy; flavio.cognigni@uniroma1.it (F.C.); marco.rossi@uniroma1.it (M.R.); 3 Institute for Complex Systems, National Research Council (ISC-CNR), Sapienza University of Rome, P.le A. Moro 2, 00185 Rome, Italy; roberta.angelini@cnr.it; 4 Physics Department, Sapienza University, P.le Aldo Moro 2, 00185 Rome, Italy; 5 Department of Chemistry, Sapienza University of Rome, P.le Aldo Moro 5, 00185 Rome, Italy; sara.alfano@uniroma1.it (S.A.); marianna.villano@uniroma1.it (M.V.); andrea.martinelli@uniroma1.it (A.M.); 6 Department of Biotechnology, University of Verona, Via Strada Le Grazie 15, 37134 Verona, Italy; david.bolzonella@univr.it

**Keywords:** poly(3-hydroxybutyrate-co-3-hydroxyvalerate), polylactic acid, polymer blends, rheology of polymer melts, fused deposition modelling

## Abstract

Polymeric materials made from renewable sources that can biodegrade in the environment are attracting considerable attention as substitutes for petroleum-based polymers in many fields, including additive manufacturing and, in particular, Fused Deposition Modelling (FDM). Among the others, poly(hydroxyalkanoates) (PHAs) hold significant potential as candidates for FDM since they meet the sustainability and biodegradability standards mentioned above. However, the most utilised PHA, consisting of the poly(hydroxybutyrate) (PHB) homopolymer, has a high degree of crystallinity and low thermal stability near the melting point. As a result, its application in FDM has not yet attained mainstream adoption. Introducing a monomer with higher excluded volume, such as hydroxyvalerate, in the PHB primary structure, as in poly(hydroxybutyrate-co-valerate) (PHBV) copolymers, reduces the degree of crystallinity and the melting temperature, hence improving the PHA printability. Blending amorphous poly(lactic acid) (PLA) with PHBV enhances further PHA printability via FDM. In this work, we investigated the printability of two blends characterised by different PLA and PHBV weight ratios (25:75 and 50:50), revealing the close connection between blend microstructures, melt rheology and 3D printability. For instance, the relaxation time associated with die swelling upon extrusion determines the diameter of the extruded filament, while the viscoelastic properties the range of extrusion speed available. Through thoroughly screening printing parameters such as deposition speed, nozzle diameter, flow percentage and deposition platform temperature, we determined the optimal printing conditions for the two PLA/PHBV blends. It turned out that the blend with a 50:50 weight ratio could be printed faster and with higher accuracy. Such a conclusion was validated by replicating with remarkable fidelity high-complexity objects, such as a patient’s cancer-affected iliac crest model.

## 1. Introduction

The increasing public awareness of the environmental implications of many plastic-based consumer items and traditional manufacturing techniques prompted the quest for sustainable and renewable materials and less ecologically impactful production technologies [1]. According to the World Commission on Environment and Development, the industrial sector is one of the most critical areas where radical change is required to achieve sustainability [2]. Such a goal entails switching to more resource-efficient production methods to reduce the input and output intensity per unit of gross domestic product to avert the repercussions of climate change, natural resource depletion, and ecological system disruptions.

New technologies, such as additive manufacturing (AM), could help reduce the carbon footprint [3,4,5]. The absence of waste products, as in a subtractive process, is an inherent feature of this method that allows for material savings. Building a product wherever it is most needed using AM is now possible [6]. As a result, transportation-related carbon dioxide emissions are reduced [7]. Deposition Modelling (FDM) is widely used as an additive manufacturing technique. A thermoplastic polymer filament is extruded through a nozzle at a temperature above the glass transition or melting point and deposited in successive layers to construct an object. The advantages of FDM over conventional manufacturing, such as flexibility in part design, mould-free production, and easy access to intricate components, have contributed to the technology’s rapid ascent to the forefront of the manufacturing industry. FDM can potentially streamline the manufacturing process for lightweight auto and aerospace parts. Furthermore, waste material can be transformed into final products with low amounts of energy and chemical treatment [8]. Three-dimensional printing employing biopolymers is the next logical step in making the process more sustainable. Unlike traditional plastics, biopolymers have several advantages, including a lower carbon footprint and more waste management options. In general, bioplastics are bio-based or biodegradable or are materials that can present both features, and they are increasingly used in many different fields, such as packaging, agriculture, consumer electronics, textiles, and automobiles. The need for innovative biopolymers in various emerging markets is fuelling this expansion and the evolution of plastics.

In general, thermoplastic materials derived from renewable sources used for FDM are currently limited to amorphous polymers or those with low crystallinity. This is mainly due to their low polymer shrinkage and warpage, which is critical to achieve high accuracy during printing.

Among the most known biopolymers, polylactic acid (PLA) and poly(hydroxyalkanoates) (PHAs), which are both bio-based and biodegradable, gained a lot of interest in the 3D-printing field [9]. PLA is a bio-based, biodegradable and biocompatible polymer [10]. The production starts with the conversion of agronomical feedstocks (corn, potatoes, sugar beet) into dextrose, whose fermentation produces lactic acid. The latter is transformed into lactide in the presence of a catalyst. After purification, lactide is used for PLA production by polymerisation. PLA is a highly versatile aliphatic polyester with excellent barrier properties. It is available in high-performance grades that are a good substitute for polystyrene (PS), polypropylene (PP), and acrylonitrile–butadiene–styrene (ABS) in more demanding applications. PLA tensile properties vary depending on whether it is annealed or oriented, as well as its degree of crystallinity.

As for PHAs, these are a family of polyesters synthesised by over 300 species of microorganisms as granules in the cytoplasm to be used as carbon and energy reserves [11]. Currently, over 150 monomers of R-hydroxyalkanoic acids have been identified. This wide variety of monomers yields PHAs encompassing a broad spectrum of thermochemical properties, which can be tailored to a wide range of applications. The most common PHAs are the poly(hydroxybutyrate) (PHB) homopolymer and the poly(hydroxybutyrate-co-hydroxyvalerate) (PHBV) copolymer, whose physical and mechanical properties strongly depend on the HV content [12,13]. Two significant drawbacks prevent the widespread use of PHB and PHBV with low HV content as a commodity. First, the polymers can be rigid and become progressively more brittle over time. Secondly, melting temperatures (around 180 °C for PHB) are close to degradation temperature, resulting in poor processing properties, not ideal for 3D-printing applications. Thus, PHB and/or PHBV were blended with other biopolymers like PLA and plasticising agents to improve processability. The development of PLA/PHA blends has been the subject of extensive studies, and a range of applications, including in the biomedical field, were proposed [14,15]. For instance, Mencik et al. [16] investigated the role of different plasticisers on warping, thermal stability, and long-term mechanical stability of printed dumbbell specimens made from various PLA/PHBV blends. Another research focus was the study of plasticiser diffusion within the matrix. Fuentes et al. [17] incorporated a styrene–acrylate copolymer with oxirane moieties as a chain extender in PLA/PHBV blends to enhance printability and printed products’ mechanical characteristics. To the authors’ knowledge, no papers have been dedicated to determining the printability landscape of commercial PLA/PHBV blends.

This article is divided into three sections: the first includes morphological and thermal analyses of the blends and a thorough rheological characterisation of the melts to extract information critical to the printing phase. In the second, we investigated printing conditions in detail to determine the optimal printing parameter, outlining each blend’s advantages and limits. In the third section, we evaluated the mechanical properties of printed specimens and the potential of the better-performing blend in the high-fidelity realisation of geometrically complex objects.

The characterisation strategy reported in this article may provide helpful feedback for readers involved in the development of thermoplastic materials for FDM that employ increasing amounts of PHAs.

## 2. Experimental Section

### 2.1. Materials

Helian Polymers B.V. (Belfeld, The Netherlands) manufactured the two spools used in the 3D-printing experiments. They were composed of blends of poly(lactic acid) (PLA) and poly(hydroxybutyrate-co-hydroxyvalerate) (PHBV, with a hydroxyvalerate content lower than 5%) in two different weight proportions: 50/50 and 25/75. In the following, we will refer to them as PLA/PHBV50:50 and PLA/PHBV25:75.

### 2.2. Methods

#### 2.2.1. Three-Dimensional Printing

Three-dimensional printing was performed with a home-built three-axis printer with an available space for printing of 200 × 200 × 200 mm^3^. AutoCAD 2020 version 23.1 was used to create 3D virtual models. The 3D models were exported in rapid prototyping format (.stl) and uploaded to Ultimaker Cura software 4.0, where a slicing algorithm was used to generate G-code. The FDM printer was fed with 1.75 mm blend filaments containing, respectively, (i) PLA/PHBV50:50 and (ii) PLA/PHBV25:75. The nozzle diameters used in the printing experiments were 0.4, 0.5, 0.6, and 0.8 mm, and the nozzle temperature was set at 180 °C or 190 °C. For all layers, the printing speed was kept between 10 and 50 mm/min. Layer height was adjusted to 0.2 mm. The 3D-mesh infill percentage was set to 100% to achieve the best mechanical properties.

#### 2.2.2. Study of the Effect of the Extrusion Speed on Extruded Filament Diameter

The experimental procedure followed to study the influence of extrusion speed on filament diameter is described in ref. [18]. Extrusion speeds ranging from 10 to 3000 mm/min were investigated, along with four nozzle diameters of 0.4, 0.5, 0.6, and 0.8 mm. The extrusion temperature was kept constant at 180 °C. The program Pronter Face 2.0.1 was used to set the extrusion speed and filament length. To minimise the effect of gravity, only the first 20 cm of the free-formed extruded filament were considered for diameter measurement. A digital comparator (Mitutoyo ID-S) was used to measure the filament diameter at fixed sampling intervals of 1 cm. The filament diameters were calculated for 20 sampling points. All data are expressed as the mean ± standard deviation and analysed using diagnostic Q–Q plots and a Shapiro–Wilk normality test. The data all fit into a normal distribution.

#### 2.2.3. Study of the Influence of Printing Parameters on Shape Fidelity and Resolution

The printability tests were designed to optimise printing settings to enhance the structural homogeneity, shape and dimension fidelity. The printing parameters that were considered include extrusion speed, flow percentage, number of layers, specimen length, and deposition plate temperature. Specimens with different geometries (cube, parallelepiped, cylinder and dumbbell) and dimensions (length, number of layers) were printed. Qualitative and quantitative assessments were performed to evaluate the degree of matching with the CAD models.

#### 2.2.4. Evaluation of the Warping Degree

The warping angle was measured on specimens with definite dimensions (length, number of layers) and geometries (laminas, dumbbells). The definition of warping angle is depicted in Figure S1. A quantitative evaluation was derived using the following formula:

$$\theta =2\arctan\left(\frac{2d}{h}\right)$$

### 2.3. Material Characterisation

#### 2.3.1. Thermal Analysis

DSC calorimeter (Mettler Toledo DSC 822, Milan, Italy) was used to investigate the thermal properties of the PLA–PHBV blends. Tests were performed on filaments and 3D-printed specimens of about 4–7 mg loaded into aluminium pans. The temperature program consisted of a first heating ramp from room temperature to 200 °C, a cooling to −100 °C and a second heating to 200 °C, all carried out at 10 °C min^−1^ scan rate. From the thermograms of the cooling scan the glass transition temperature at the midpoint (*T_g_*), the specific heat variation at *T_g_* (∆*C_p_*), the melt-crystallisation peak temperature (*T_mc_*) and enthalpy (∆*H_mc_*) were evaluated. In the second heating, in addition to *T_g_* and ∆*C_p_*, the cold-crystallisation peak temperature (*T_cc_*) and enthalpy (∆*H_cc_*) as well as the temperature (*T_m_*) and enthalpy of fusion (∆*H_m_*) were determined.

#### 2.3.2. Rheological Measurements

Rheological measurements were carried out with an Antoon Paar MCR102 rheometer (Rivoli, Italy) using a 50 mm diameter cone plate geometry with a gap of 0.212 mm. The rheological analyses were performed at 180 and 190 °C, considering a melting time of 20 min to ensure the blends were completely melted. Flow curves under a steady rotational regime were recorded between 0.1 and 100 s^−1^ under a continuous nitrogen flow to avoid thermal degradation.

Small amplitude oscillatory tests were performed to determine the limit of the linear viscoelastic region (at frequency *ω* = 1 Hz). Frequency sweep experiments were used to determine the viscoelastic characteristics of the two blends. Rheological parameters (storage modulus, *G*′; loss modulus, *G*″, complex viscosity, η*) were directly obtained using the manufacturer-supplied computer software (Rheocompass 1.1). The loss factor was calculated from the ratio between *G*″ and *G*′, as follows:

tan *δ* = *G*″/*G*′

where *δ* the phase shift angle ranges between 0° and 90°.

Cole–Cole graphs were obtained by plotting the imaginary viscosity (*η*″) as a function of the real viscosity (*η*′). The first normal force (*N*_1_) was calculated from the Laun formula [19].

$$\left.N_{1}\left(\phi \right)\right|=2G^{\prime }\left(\omega \right){\left\{1+{\left[\frac{G^{\prime }\left(\omega \right)}{G^{\prime \prime }\left(\omega \right)}\right]}^{2}\right\}}^{0.7}$$

where *G*′(*ω*) and *G*″(*ω*) are the elastic and loss moduli, respectively.

#### 2.3.3. Compressive and Tensile Testing of 3D-Printed Materials

Compressive and tensile properties were measured using an Instron 4502 testing machine equipped with a 10 kN load cell. Tests under compression were carried out according to the ASTM D695-15 standard [20] on printed cylinders with a diameter and height of 1 cm or cubes with a side length of 1 cm. The compressive moduli of PLA and PLA–PHBV blends were measured by single uniaxial unconfined compression. Tensile testing was performed on dog bone specimens based on ASTM D638 standard [21]. Two different printing raster angles (0° and 90°) were used to determine the effect of patterning on the tensile properties. The elastic modulus (*E*) was measured at a crosshead speed of 0.25 mm/min. At least five specimens for each sample were tested.

#### 2.3.4. Scanning Electron Microscopy

A field emission scanning electron microscope (FESEM), AURIGA ZEISS (Milan, Italy), operating at 5 kV, was used to study the internal surface morphology of the samples. Cryo-fractured samples in liquid nitrogen were mounted on aluminium stubs using a copper adhesive film and silver cement to enhance the conductivity of the specimens and improve the quality of images taken at high magnifications (×50 k). Specimens were then coated with a 20 nm thick chromium film using Q 150T ES-EMITECH sputter (Milan, Italy). Images were recorded by operating the FESEM in the InLens modality (secondary electrons).

#### 2.3.5. Computed X-Ray Microtomography (μCT)

X-ray Microscopy (XRM) is a non-destructive characterisation that provides 2D/3D information about the specimen’s microstructure. Since X-rays can penetrate the matter, XRM reveals hidden and internal details of the sample without cutting or damaging the sample before or during the analysis. A typical experimental setup is composed of (a) the source that produces the X-ray beam; (b) the sample stage that allows the rotation of the sample during the scan and ensures its stability; (c) the detector (CCD or CMOS) which collects the transmitted X-ray beam. A single projection is acquired at every rotation angle and at the end of the process a set of projections is fed into a tomographic reconstruction algorithm to obtain a digital 3D model of sample.

Acquisition was performed on models of a shaft support and an iliac crest using an Xradia Versa 610 with the following parameters: voltage 50 kV, power 4.50 W, objective ×0.4, exposure time 3/5 s.

Since the horizontal dimensions of the sample exceeded the maximum Field Of View (FOV) achievable in the Normal Mode, these experiments were carried out adopting the Wide Field Mode (WFM) available for the X-ray microscope ZEISS Xradia Versa 610 (ZEISS, Milan, Italy). WFM allows the horizontal maximum FOV to be doubled by shifting the sample to the right and then to the left, acquiring two adjacent projections that are automatically stitched by the software at the end of data acquisition. This allowed us to investigate the entire sample non-destructively.

The scans were set up for both samples using the Scout-and-Scan Control System (Version 16.1.13038.43540) from Carl Zeiss (Milan, Italy).

## 3. Results and Discussion

The successful 3D printing of a thermoplastic polymer, as measured by shape fidelity and resolution, is dependent on both the intrinsic physical properties of the polymeric blend and the printing conditions. Glass transition and melting temperatures, mutual miscibility of the polymeric components in the blend and viscoelasticity are essential physical properties that must be evaluated before proceeding to the printing stage. Printability experiments entail configuring printing parameters such as extrusion rate, printing speed, infill percentage, flow percentage, collection stage temperature, and extrusion temperature. It is worth stressing that print settings are inextricably linked to the blends’ physical properties; hence, the first section of the work is devoted to the characterisation of the latter.

### 3.1. Blends’ Thermal Properties

The literature on the miscibility of PLA and PHBV blends is contradictory, with reports of partial miscibility [22,23,24], complete immiscibility [25,26], and/or mutual influences between the two polymers [27]. The results are also influenced by the composition of the blends and the processing conditions used for the sample preparation. Therefore, the DSC experiments were conducted on blends and neat PLA filaments as well as 3D-printed objects. The first heating scan was performed to characterise the initial material behaviour, while the subsequent cooling and heating scans were conducted to interpret the observed transitions. The DSC traces obtained from filaments and printed objects did not exhibit substantial variations, and the results of filament samples will be presented in the following section.

**Figure 1:**
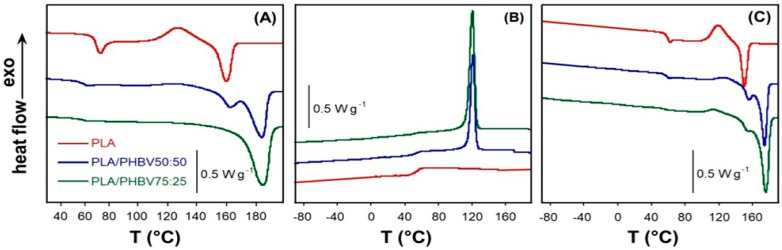
DSC thermograms of neat PLA, PLA/PHBV50:50 and PLA/PHBV25:75 blends: (**A**) first heating; (**B**) cooling; and (**C**) second heating scans all carried out at 10 °C min^−1^.

The DSC thermograms of the two blends and neat PLA, provided as a reference, acquired in the first heating, cooling and second heating scans, all carried out at 10 °C min^−1^, are shown in Figure 1A, Figure 1B and Figure 1C, respectively. The results of the data elaboration are reported in Table 1.

It is well-known that PLA is a polymer with a very low crystallisation rate and remains nearly completely amorphous when cooled rapidly from the melt. As a consequence, it shows a well-defined glass transition spanning between 52 and 58 °C and a specific heat variation (∆*C_p_*) normalised with respect to the PLA weight fraction almost equal to that of 100% amorphous sample (∆*C_p_*^0^ = 0.51 J g^−1^ k^−1^ [28]). The high PLA *T_g_* observed in the first heating scan is attributed to the superposition of the intense overshoot peak. At temperatures above the *T_g_*, both the neat PLA and the PLA fraction in the PLA–PHBV50-50 blend undergo cold crystallisation at *T_cc_* = 119 °C and *T_cc_* = 116 °C, respectively.

The absence of the glass transition inflection at around 0–5 °C in the blends’ DSC curves suggests that the PHBV phase in both starting blends is semi-crystalline.

The enthalpy of cold crystallisation of PLA–PHBV50-50 (∆*H_cc_* =5 J g^−1^), well below that expected from the value found for the neat polymer, evidences the hindering effect of the crystalline PHBV phase on the PLA ordering. Therefore, because of the low PLA content in PLA/PHBV25:75, the cold crystallisation process is nearly absent. The PLA melts at 152 °C in the neat sample and at 155 °C in the PLA/PHBV50:50 blend while the process is absent or partially hidden in PLA–PHBV25:75 by the large endothermic peak at the highest, due to the melting of the PHBV fraction in the blends.

Considering that the contribution of PLA to the overall enthalpy of fusion should be approximately ∆*H_cc_*≈∆*H_m_* =5 J g^−1^, the ∆*H_m_* of PHBV phase in PLA–PHBV50-50 is 50 J g^−1^, which is 100 J per gram of polymer. The PLA/PHBV25:75 melting peak did not contribute to the PLA; therefore, an enthalpy of fusion of 95 J per gram of PHBV could be inferred. Then, the PHBV fraction in PLA/PHBV25:75 has about the same crystallinity as PLA–PHBV50:50. In the cooling process carried out at 10 °C min^−1^, the melt crystallisation of the PHBV fraction in the two blends occurs at the same temperature (*T_mc_* = 120–121 °C) and involves nearly equal normalised enthalpies while PLA does not crystallise. In the subsequent heating ramp, the *T_g_* of PLA shows a slight increase and, differently from the first scan, the PLA fraction in PLA/PHBV25:75 cold crystallises at the lowest *T_cc_*, presumably because of the nucleating effect of the pre-existing highly ordered PHBV, as suggested by various authors [27,29,30,31]. Moreover, a significant increase in the overall ∆*H_m_* is observed in the blend with higher PHBV content due to an increase in the crystallinity of this polymer with respect to that found in the first scan.

In summary, the DSC results suggest that PLA and PHBV are immiscible polymers, although they highlight that the thermal behaviour of the blends is influenced by both the interaction between the two polymers and by the thermal treatment they are subjected to. Moreover, the DSC data indicate that at room temperature and under the explored conditions, the PLA fraction exists in the amorphous state and PHBV in the semi-crystalline phase.

The printing temperature in FDM is chosen just above the melting point to ensure that the extruded filament retains its cylindrical symmetry without flattening and that overhanging structures do not sag. The extrusion temperature was initially set to 176 °C; however, the too-high melt viscosity hindered continuous filament extrusion. The minimum extrusion temperature for successful continuous filament extrusion was determined to be 180 °C. To find the ideal extrusion temperature, we investigated the rheological parameters of the two blend melts at 180 and 190 °C.

### 3.2. Scanning Electron Microscopy Characterisation (SEM)

**Figure 2:**
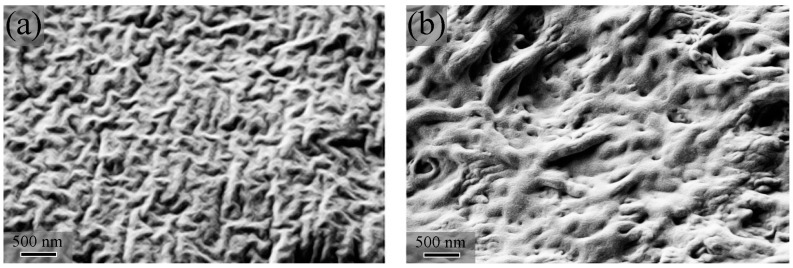
SEM micrographs of cryogenically fractured cross-sections of PLA/PHBV blends: (**a**) PLA/PHBV50:50; (**b**) PLA/PHBV25:75.

Figure 2 shows the SEM images of the cryogenically fractured surfaces of blend filaments. Both the micrographs display a ribbon-like structure of different dimensions and orientation degrees, consisting of two interpenetrated, i.e., co-continuous phases. A similar morphology was reported by Lee et al. [32], who studied melt-spun fibres in which PLA was selectively removed by solubilisation in THF. Brutting et al. [33] described the evolution of the morphology of PLA/PHBV blends with a composition from 90/10 up to 60/40. They observed that by decreasing the PLA content the droplets of the dispersed PHBV phase become larger and their shape changes from spherical to elongated ellipsoids. However, although the thermal characterisation of PLA/PHBV50:50 and PLA/PHBV25:75 evidenced that the two polymers are not miscible, Figure 2b shows that the interface between PLA and PHBV polymers, especially in PLA/PHBV25:75, was not clearly observed. A poorly defined phase boundary region was also reported by Liu et al. [34] and Zembouai et al. [27] on melt mixed blends. We can presume that the PHBV polymer, which crystallises upon cooling at higher temperatures than PLA glass transition, was wrapped in the PLA vitrified amorphous phase. The lack of well-defined boundaries between the two phases may be due to the partial mutual solubility of the two polymeric components.

### 3.3. Rheological Characterisation

**Figure 3:**
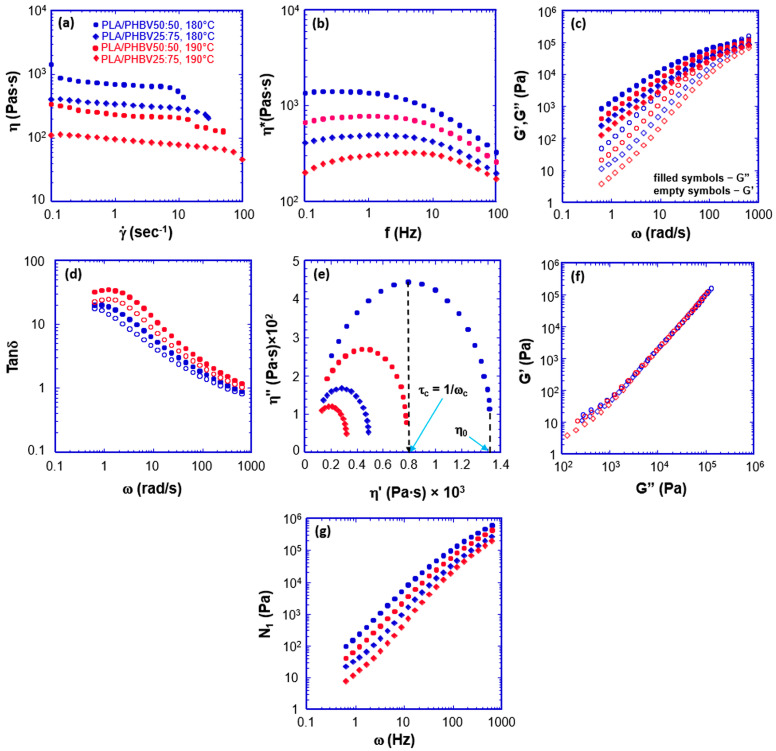
Rheological measurements of PLA/PHBV50:50 and PLA/PHBV 25:75 blends: (**a**) the effect of shear rate and temperature on the shear viscosity; (**b**) complex viscosity versus frequency of oscillation, (**c**) storage (*G*′, empty symbols) and loss moduli (*G*″, filled symbols) and (**d**) loss factor (tan *δ*) versus angular frequency. (**e**) Cole–Cole plots of imaginary (*η*″) versus real viscosity (*η*′) (the dotted vertical line shows an example of the extrapolation from a curve maximum to the X axis for the determination of the relaxation time (*τ_c_*)) and (**f**) of the storage modulus versus the loss modulus. (**g**) First normal stress (*N*_1_) as a function of angular frequency (*ω*).

Investigating the melt’s rheological behaviour provides valuable insight into the printing process’s influencing parameters; therefore, a series of rheological measurements were performed. The discussion of the rheological result assumes that the two phases, as revealed by SEM and DSC data, persist in the melted state. As it will be demonstrated, this assumption allows a coherent and unified frame for all the rheological results. The shear viscosity of the two blends was investigated as a function of shear rate at two distinct temperatures, 180 °C and 190 °C, as shown in Figure 3a. The blend with higher content of PHBV (PLA/PHBV25:75) at both the temperatures investigated exhibits the lowest viscosity over the full range of shear rate. This result is in accordance with the work by Li et al. [32], who recorded the shear viscosity of a series of PLA/PHBV blends from pure PLA to pure PHBV. Neat PLA has the highest viscosity, while neat PHBV has the lowest. PLA/PHBV50:50 at 180 °C and, to a lesser extent, at 190 °C exhibit a trend characterised by a step decrease at the extremities of the flow curves. The middle portion, on the other hand, experiences a minor decrease as the shear rate increases. The two-phase nature of the blends may explain such behaviour. As revealed by SEM micrographs, the co-continuous morphology consists of long, undulated ribbons of PHBV encased in the PLA phase. Under low shear conditions, the PHBV-rich phase starts flowing first because of its lower viscosity and ribbons tend to align along the imposed flow lines. Any distortion of ribbons from the equilibrium shape causes an increase in capillary pressure that opposes deformation. The PLA phase also starts flowing as the shear stress equals the capillary pressure. Thus, the subsequent moderate decrease in viscosity can be ascribed to the combined flow of PHBV and PLA rich-phases. The second step at the high shear rate end is likely the result of PHBV and PLA macromolecules flowing along the flow lines.

In PLA/PHBV25:75, the first initial step is less pronounced and extends to a higher shear rate until ~10 s^−1^. This may be due to the larger aspect ratio of the PHBV-rich phase domains (Figure 3b), which can deform significantly before capillary pressure comes into action and causes the PLA phase to flow. The above data interpretation is in accordance with previous theoretical predictions and experimental findings [35].

The increase in temperature to 190 °C causes a decrease in the interfacial tension and the surface elastic response [36,37], resulting in smoother transitions between the regimes with the flow curve at a low shear rate exhibiting a pseudo-Newtonian behaviour. 

The flow behaviour of the melts can be analysed from a distinct perspective by plotting the complex viscosity as a function of frequency. The results are displayed in Figure 3b. The most relevant feature emerging from the analysis of complex viscosity curves is the presence of a maximum. In PLA/PHBV50:50 the maximum is hardly discernible and is at 0.27 Hz. After the maximum, the complex viscosity drops significantly, a behaviour typical of pseudoplastic systems. At 190 °C, the maximum becomes more prominent and shifts to 1.93 Hz. We observed the same trend with PLZ/PHBV25:75, with the maxima becoming more prominent and shifting to higher frequencies. For instance, at 180 °C, the maximum falls to 1.39 Hz, while at 190 °C the maximum is shifted to 3.73 Hz.

Other authors have observed such behaviour. In oscillatory experiments, the input energy is stored in part as elastic energy, and part is dissipated. Complex viscosity is a measure of the dissipated energy. The presence of a maximum indicates that at low frequency (on the left side of the maximum), the melts display a more elastic behaviour [26,38,39,40]. Under oscillatory stress, the interface between the two phases deforms, assuming an enhanced anisotropic state with the interfacial area becoming larger. Capillary pressure opposes deformation. Thus, the interface behaves to some extent as an elastic membrane. The absence of a significant maximum in PLA/PHBV50:50 trace at 180 °C indicates that the interface is relatively rigid and does not undergo substantial deformation during an oscillation period. This is in accordance with the ribbon’s high aspect ratio, as evidenced by SEM micrographs (Figure 2a). As stated above, increasing temperature decreases the interfacial tension making the interface more deformable and capable of storing a higher % of elastic energy. PLA/PHBV25:75 at 180° and 190 °C exhibit more pronounced maxima because of the lower aspect ratio. During the oscillation period, ribbons deform to a larger extent and store a higher amount of the input energy.

An important aspect to evaluate in view of 3D printing is the determination of the viscoelastic properties of the filaments at the indicated temperatures. A certain degree of viscoelasticity guarantees that the extruded filament can stretch without rupturing, even at high extrusion speeds. Figure 3c reports the elastic (*G*′) and loss (*G*″) moduli as a function of angular frequency (*ω*). *G*″ predominates over *G*′ in almost all the frequency ranges explored. However, in the case of PLA/PHBV50:50, at 180 and 190 °C, the cross-over frequency is observable at 45 and 70 Hz, respectively, while for PLA/PHBV25:75 is out of the frequency range. This means that PLA/PHBV50:50 relaxes faster than PLA/PHBV25:75, indicating a more pronounced solid-like behaviour. Further evidence of the more pronounced solid-like behaviour of PLA/PHBV50:50 comes from Figure 3d analysis, where the loss factor, tan *δ*, is plotted as a function of *ω*. PLA/PHBV50:50 traces are constantly below PLA/PHBV25:75 ones and cross the value of 1 at the very high-frequency end. A closer examination of the loss factor curves reveals additional information. PLA/PHBV25:75 at 180 and 190 °C exhibits a small maximum at about 0.19 Hz. Thus, for frequencies lower than 0.19 Hz, the melts have slightly more solid-like behaviour than at frequencies on the right side of the maximum. Such results agree with complex viscosity measurements.

Another interesting aspect worth investigating is the blend’s degree of heterogeneity. A useful plot that aids in evaluating such an aspect is the Cole–Cole plot, where the imaginary viscosity (*η*″) is plotted as a function of real viscosity (*η*′). This representation gives information on the relaxation processes occurring in a multiphase system. It can also be helpful to predict the compatibility of a blend. A semi-circular shape suggests a homogeneous blend, while consistent deviations from such a symmetry indicate the presence of heterogeneities [41]. Furthermore, the Cole–Cole plot allows the determination of rheological parameters such as the zero-shear viscosity (*η*_0_) as the extrapolation to the x-axis (*η*′ at *η*″ = 0) and the relaxation time (*τ*) as the inverse of the frequency at which maximum occurs. Figure 3d shows the Cole–Cole plots of the two blends at 180 and 190 °C. All traces have a parabolic profile, indicating that the blends at all temperatures are homogeneous in the length scale explored by rheology. The PLA/PHBV50:50 curves (180 and 190 °C) form larger arcs than PLA/PHBV25:75, indicating broader relaxation times and higher zero-shear viscosities. Consequently, the former blend is less flowable and requires longer times to respond to the imposed stress. Table 2 reports the blends’ zero shear rate viscosities and relaxation times at the two temperatures.

The validity of the observation based on Cole–Cole traces was further supported by *G*′ versus *G*″ plots on a logarithmic scale to investigate the homogeneity of the blends further. The curves for a homogeneous polymer system are not dependent on the temperature and are generally linear [42]. From Figure 3e it is possible to observe that all traces fall on a single curve. Minor deviations occur at the low-frequency end. It is likely that a low frequency, imposed flow and subsequent ribbon deformation and alignment cause temporary anisotropies.

Finally, we determined the first normal stress by using the Laue formula. Normal forces are generated during shearing and act perpendicularly to the direction of shear. Under shear, ribbons and macromolecular chains tend to align along the flow lines, causing a drop in entropy. First normal stress is a measure of the tendency of the ribbons/polymeric chains to regain the lost entropy. Figure 3f shows that in all angular frequency ranges explored, the first normal stresses referring to PLA/PHBV50:50 at both temperatures are constantly higher than PLA/PHBV25:75. Normal stresses play a fundamental role in dye swelling upon extrusion.

In thermoplastic polymer 3D printing, the extrusion temperature is set close to the melting temperature to ensure the rapid solidification of the deposited filament and shape retention. The shear and complex viscosities at 180 °C are in the range of ~1000 Pa s^−1^ and are not too high to cause inconsistent flow or jamming at the nozzle [43]. Therefore, the 3D-printing experiments described in the following sections were carried out at 180 °C.

## 4. Three-Dimensional Printing

### 4.1. Filament Diameter vs. Speed of Extrusion

The diameter of the extruded filament is crucial in affecting the printed products’ quality and degree of resolution. In this regard, two opposing requirements must be met: time efficiency and printing resolution. Large objects demand a long printing time; hence, faster printing speeds allow for a faster total fabrication process. Extrusion of molten plastic materials, on the other hand, experiences a phenomenon known as die swell, manifested as diametric inflation of the extrudate while transitioning from a confined flow to a free flow. This phenomenon is driven by ribbon alignment (as in this specific case), macromolecule disentanglement and orientation along the flow lines [44]. The process involves ordering the fluid’s structure, resulting in a decrease in entropy proportional to the shear stress. Upon extrusion, both aligned ribbons and macromolecules relax, and an entropy gradient develops across the filament radial direction, originating the normal forces at the origin of the dye swell.

**Figure 4:**
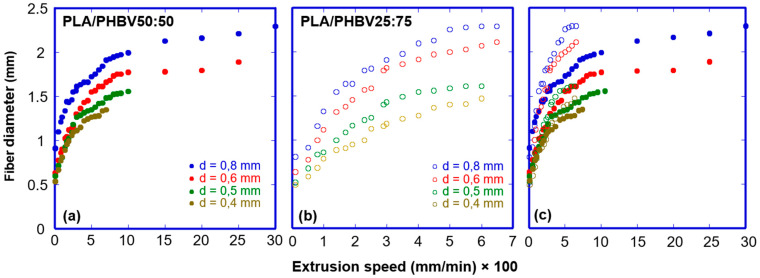
Dependence of filament diameter on extrusion speed and nozzle diameter at 180 °C for PLA/PHBV 50:50 (**a**) and PLA/PHBV 25:75 (**b**), with (**c**) showing the overlap of graphs for comparison.

It is, therefore, critical to define the range of accessible extrusion speed without compromising filament integrity and uniformity and to design calibration curves relating filament diameter to extrusion speed and nozzle diameter. Figure 4a,b show the results for PLA/PHBV50:50 and PLA/PHBV25:75, respectively.

All curves show an initial rapid increase and tend to plateau at elevated extrusion velocities. This behaviour aligns with prior research [44,45] that establishes a direct relationship between the flow shear stress and the extent of die swell in thermoplastic polymers.

When extrusion speeds exceed the upper limit, the extruded filaments experience a phenomenon known as buckling [46,47]. The high extrusion speed results in insufficient residence time of the blend in the liquefier, leading to inadequate softening and the persistence of high viscosity and shear stress. Consequently, the extruder motor must apply an increased torque to extrude the material from the nozzle. Buckling occurs in the filament when the applied force surpasses its compressive modulus. The extrusion process experiences intermittent behaviour when the material remains immobilised at the convergence zone of the extruder, allowing it to accumulate sufficient energy for melting.

One notable observation from the comparison of Figure 4a,b is the significantly larger range of extrusion speed exhibited by PLA/PHBV50:50 compared to PLA/PHBV25:75 across all the nozzle diameters employed. As an illustration, while employing a nozzle diameter of 0.8 mm, a consistent filament is produced at extrusion speeds of up to 3000 mm/min for the PLA/PHBV50:50 blend, the maximum limit is 700 mm/min for the PLA/PHBV25:75 blend. The relationship mentioned above remains consistent for the other nozzle diameters; however, the range of extrusion speeds that can be achieved decreases correspondingly as the nozzle diameter decreases. The ability to use PLA/PHBV50:50 at higher extrusion speeds is most likely due to its more pronounced viscoelastic characteristics, which allow filament stretching and compression (Figure 3c) without rupturing and buckling.

The two sets of curves are juxtaposed in Figure 4c. PLA/PHBV25:75 filament has consistently larger diameters than PLA/PHBV50:50 filament at the same extrusion speed and using the same nozzle, showing that the former blend dye swells to a greater extent during extrusion. This result contrasts with the first normal stress data (Figure 3g), which shows that PLA/PHBV50:50 experiences a stronger driving force to filament inflation when sheared. First normal stress is only one of the physical parameters involved. The relaxation time (Table 2), which is significantly smaller in the case of PLA/PHBV25:75 than in the case of PLA/PHBV50:50 at both studied temperatures, is another crucial parameter. Because of the longer relaxation times, PLA/PHBV50:50 filament shows a minor degree of dye swell in the time frame between extrusion and solidification. PLA/PHBV25:75, on the other hand, swells to a greater extent because of the relatively short relaxation time, resulting in larger diameter fibres.

As illustrated in Figure 4, the wide extrusion rate range accessible for both blends potentially enables the rapid construction of large items. To verify if this is true, a parallelepiped specimen (Figure S2a) as a function of deposition speed was printed. The resulting structures were evaluated in terms of shape, dimension fidelity and absence of flaws.

A close match between the CAD model and printed specimens in the range of up to 50 mm/min (Figure S2b), regardless of nozzle diameter and blend type, was found. Above 50 mm/min, some defects, whose size depended on the nozzle diameter, were noticed. The 0.8 mm diameter nozzle produced a poorly defined specimen. No imperfections were found in the 70–120 mm/min deposition speed range with a 0.4 mm diameter nozzle. On the contrary, the parallelepipeds printed with 0.5 and 0.6 mm diameter nozzles exhibited passing through holes whose diameters increased proportionately to deposition speed and nozzle diameter (Figure S2c). Figure S2d,e show two examples of parallelepipeds with visible holes.

Unlike unfettered extrusion, the deposition process is constrained by its surroundings. The deposited layer adheres to the previous layers and is dragged in the movement’s direction. The free extrusion of the filament is intermittent, as illustrated in 4.1, because the material is not sufficiently melted (low residence time inside the liquefier). The same effect occurred during the printing of sample parts, resulting in the presence of extrusion speed-dependent voids.

Based on the analysis conducted, the exploitable range of extrusion speed falls within the range of 10–50 mm/sec, regardless of the nozzle diameter employed.

### 4.2. Effect of Flow Percentage (%Flow)

Percentage flow is an adjustable parameter within the slicer program that can be modified to deliver the filament required to fill the volume within the designed item. The amount of filament to be extruded is calculated when the slicer generates the. Gcode file. We previously discussed how dye swelling occurs during extrusion in the polymeric melt filament. As a result, %flow must account for this occurrence to ensure products are compliant with the theoretical design requirements. To this end, we drew calibration curves that relate the linear dimensions of simple geometrical objects (cubes, cylinders, and dumbbells) to the %flow. Such calibration curves are useful in practice because they compensate for die swelling and guarantee a better match between the design and the actual item dimensions.

**Figure 5:**
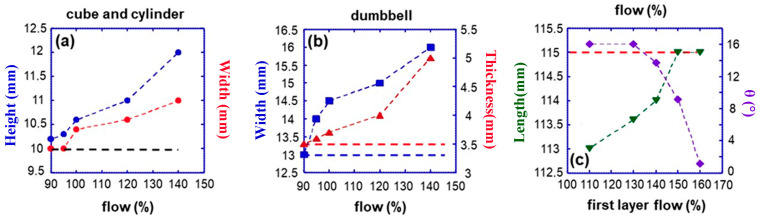
Nominal dimensions of the printed specimens using the blend PLA/PHBV50:50. Dependence of samples dimension on % flux: (**a**) cube (10 × 10 × 10 mm^3^) and cylinder (d = 10 mm, h = 10 mm), (**b**) dumbbell (115 × 13 × 3 mm^3^). (**c**). Influence of initial layer flow rate % on dumbbell length and degree of warpage. The horizontal dotted lines mark the expected dimension according to the CAD models.

Figure 5a reports the PLA/PHBV50:50 blend results. The linear dimension increases with the flow percentage for both the cube and cylinder specimens. For example, with a %flow of 100%, the specimens’ height and width depart from the predefined values by 4 and 6%, respectively, and this discrepancy grows as the %flow increases. To account for deviation, the %flow must be reduced below 100%, precisely to 90% (Figure 5a). At this value, there is good agreement with the designed dimensions. The dumbbell specimen yielded similar results in terms of thickness and width. The optimal %flow was 90% (Figure 5b).

Regarding dumbbell length, we observed a modest degree of specimen detachment and arching from the printing plane, decreasing the specimen’s designed length. This phenomenon is referred to as warping [48,49,50], which is a deformation or distortion of printed items, especially large or flat objects. Warping is a typical problem in FDM caused by the uneven distribution of stress during printing and could be influenced by crystallisation. In fact, during inhomogeneous cooling across the material, different and uncontrolled crystallisation rates can arouse internal stresses and strains. These unequal tensions can cause warping when the materials try to relieve the stress build-up and establish a more relaxed state. Building a larger initial layer to enhance the area of contact with the bottom deposition plane is one technique for reducing the influence of warping. This is accomplished by simply raising the percentage flow, which, as previously stated, generates an increase in nominal linear dimension, mainly along the length of the dumbbell specimen. Figure 5c demonstrates that increasing the %flow of the initial layer to 160% cancels the warping effect with an almost full recovery of the designed length. In summary, the optimal percentage flow values for dumbbell specimens are 160% for the first layer and 90% for subsequent layers. 

**Figure 6:**
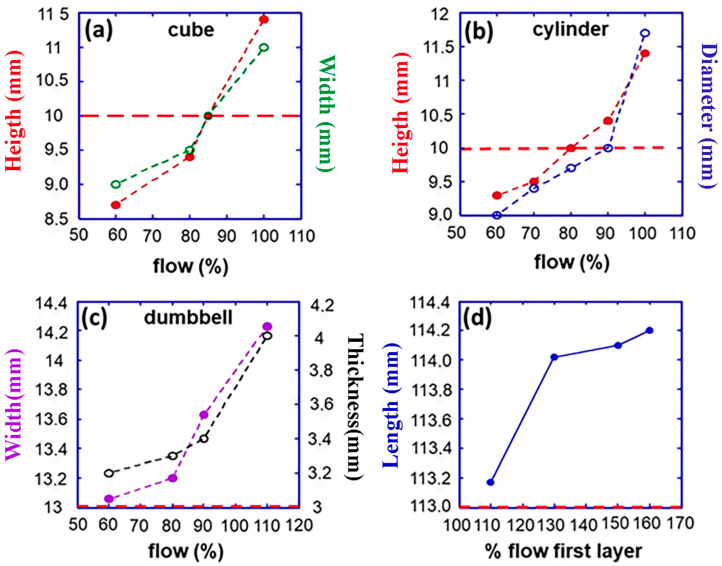
Dependence of printed specimen dimension on % flow: (**a**) cube, (**b**) cylinder, (**c**) dumbbell, (**d**) influence of flow % of the first layer on dumbbell length. All specimens are obtained from PLA/PHBV50:50. The red dotted horizontal lines mark the expected dimension according to the CAD models.

**Figure 7:**
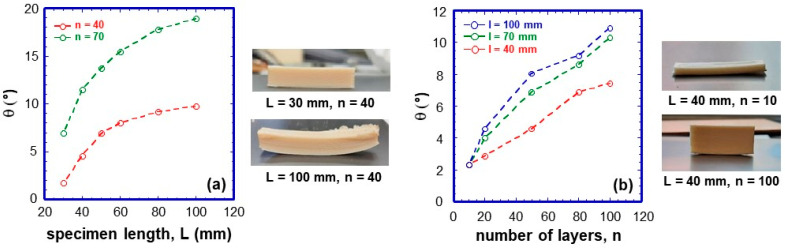
(**a**) Influence of specimen length (*L*) for two different number of layers and (**b**) specimen number of layers (*n*) for three different specimen lengths on warping (*θ*). Specimens were printed using the PLA/PHBV25:75blend. Deposition plate temperature: 25 °C; specimen width: 10 mm. On the right side of the plots, specimens’ photographs illustrate the influence of length and number of layers on warping angle. Photographs are not in scale.

In contrast to PLA/PHBV50:50, where the geometry of the printed specimen does not affect the optimal %flow, the blend PLA/PHBV25:75 required determining the optimal %flow for each specific geometrical shape. In the case of cubes (Figure 6a), it was determined that the ideal %flow was 85%. Similarly, the optimal % flow for cylinders was found to be 80%. It is important to observe that the optimal %flow consistently exhibited lower values than the similar specimen derived from the PLA/PHBV50:50 blend. The observed outcome aligns with the experimental data presented in Figure 4c. The die-swell experienced by PLA/PHBV25:75 blend consistently exceeds that of the PLA/PHBV50:50 blend. Therefore, the adjustment required to account for die swell is inevitably greater. Deviations from designed dimensions are especially pronounced in the case of dumbbell specimens. The results for transverse and thickness dimensions are displayed in Figure 6c. Deviations are not negligible when the % flow exceeds 60%. Warping effects were highly prominent along the longitudinal direction, Figure 6d, and depended on deposition speed (Figure S4), specimen length (Figure 7a) and number of layers deposited (Figure 7b). Even at high deposition speeds, the increase in the % flow of the first layer was ineffective in recovering the required length (Figure S3b). To mitigate but not eliminate warping, a brim layer is deposited to enhance the contact surface with the deposition plate significantly. The warping angle of the first layer without and with brim is shown in Figure S3b as a function of %flow.

As observed, the brim layer significantly decreases warping but does not eliminate it. Thus, printing a brim layer is only a partial solution to warping. Additionally, it must be calibrated to the object dimension, meaning a time-consuming experimental setup.

### 4.3. Influence of Deposition Plate Temperature

**Figure 8:**
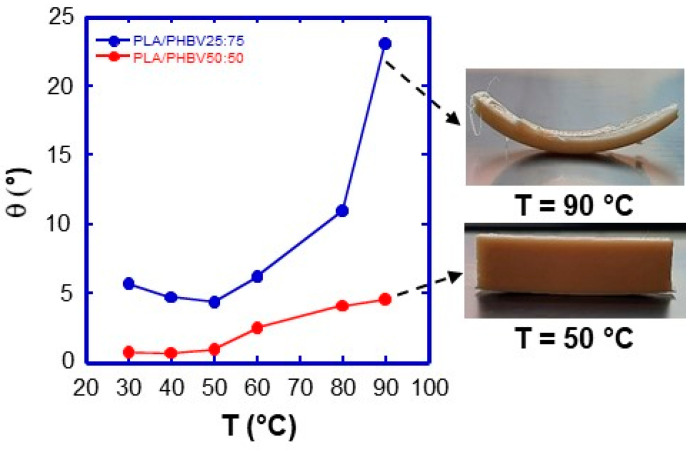
Degree of warping as a function of temperature for the two blends: PLA/PHBV27:75 and PLA/PHBV50:50. Specimens’ dimension: length: 50 mm, width: 10 mm, number of layers: 70. On the right, photographs of two lamellas characterised by different number of layer and printed at 50 and 90 °C are shown.

The deposition plate temperature is another parameter that can reduce warping. Figure 8, left shows the degree of warping, *θ*, as a function of deposition plate temperature. The PLA/PHBV25:75 blend exhibits a strong dependency, from roughly 5° to 24°. PLA/PHBV50:50, on the other hand, has a substantially lower dependence. Both blends display a minimum at 50 °C, corresponding to the *T_g_* of PLA. At temperatures below 50–60 °C, the PLA fraction rapidly vitrifies, the first layers’ geometry. At higher temperatures, the PLA polymer remains in the rubbery state, and the progressive crystallisation of PHBV can cause material shrinkage and the occurrence of compressive stresses. These effects are intensified in PLA/PHBV25:75 due to the higher PHBV content and its higher intrinsic crystallinity achieved after the blend deposition, as evidenced by the thermal characterisation.

To almost eliminate warping effects, the deposition plate temperature must be set to 50 °C and the use of the blend with a minor amount of PHBV, i.e., PLA/PHBV50:50 is recommended. Figure 8, rigth depicts the effect of the deposition stage temperature on the degree of warping of printed PLA/PHBV25:75 specimens. Even on thick samples, the warping impact is minimised by setting the temperature to 50 °C. In comparison, thinner specimens distort significantly at a plate temperature of 90 °C.

### 4.4. Mechanical Testing

A set of printed samples with varying geometries and internal structures underwent mechanical testing in both compression and tension modes. Figure S5 presents the stress–strain curves of cubic and cylindrical specimens with a 100% infill density. The mechanical behaviour of the specimens is dependent on their geometrical shape. Cylindrical specimens’ stress–strain curves exhibit a yield point but do not show stress–softening and hardening regions. The specimens have limited plasticity when subjected to stress. Cubic specimens exhibit a stress–strain response influenced by the direction of compression with respect to the printing plane (Figure S5a,b).

When compressed in a direction perpendicular to the printing plane, PLA/PHBV50:50 cubes show a yield point without a stress softening zone; meanwhile, neat PLA and PLA/PHBV25:75 display both stress softening and stiffening zones. The two blends display the opposite stress–strain behaviour when the compression direction is parallel to the printing plane. The mechanical properties’ parameters derived from the stress–strain curves are presented in Table 3 and Table 4 for the blend specimens and the specimen composed solely of PLA, which serve as a reference. Regarding cubic specimens, compression orientation has minimal impact on the mechanical properties. A clear pattern observed from the data analysis is the reduction of Young’s moduli and yield strengths as the PHBV content increases, consistent with the literature results obtained on compression or injection moulded blend samples [24,51]. Conversely, the yield strain exhibits an opposite tendency. PLA is the more rigid component, whereas PHBV has a greater excluded volume because of the bulky valerate residues. Therefore, the blend with a greater proportion of PHBV is more compliant.

Figure S6 illustrates the stress–strain curves obtained from the traction tests conducted on dumbbell samples made solely from the two different blends and PLA. The samples were printed with two distinct raster angles, 0° and 90°. The angle of the raster does not impact the mechanical properties (Table 5). Specimens with a low strain at break exhibit structural failure, most likely caused by minor defects within the structure, as evidenced by the low *σ_break_* and large data standard deviation. When subjected to stress, these imperfections serve as the starting points for crack propagation. Although precautionary measures were taken, such as disabling the Cura’s “retraction distance” function, these issues continued to exist.

### 4.5. Case of 3D Printing of Objects of Increasing Complexity

**Figure 9:**
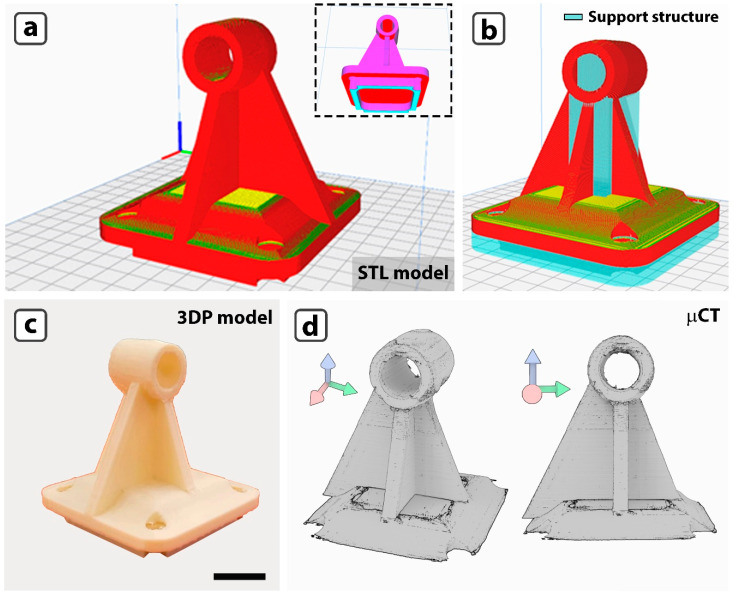
A 3D CAD model of the shaft support (**a**), and of the model including the supporting structure (shown in blue) (**b**). (**c**) photos of the printed shaft support and (**d**) computed X-ray microtomography (μCT) 3D rendering of the printed model from lateral and frontal views.

The experimental work described in the preceding sections attempted to identify the blend with the best printability, PHBV–PLA50:50, and to find the optimal printing settings. The next step is to assess its adaptability in manufacturing increasingly complex items. We concentrated on creating a technical device, a shaft holder, and a 3D model of a sacroiliac joint of a patient affected by chondrosarcoma. Figure 9a shows the shaft holder’s CAD model. It has four screw holes at the base’s vertices for securement. The presence of concave surfaces necessitates designing and installing temporary supporting structures (Figure 9b) that will be removed at the end of the printing process. The optimal printing settings are summarised in Table S1. Photographs of the printed product are remarkably faithful to the CAD model (Figure 9c). CT scans produce 3D renderings with a more precise perspective (Figure 9d) from two distinct orientations. The absence of surface defects, particularly inside the hole, is critical. This demonstrates that removing the supporting framework had no negative impact on the texture quality of the surface.

**Figure 10:**
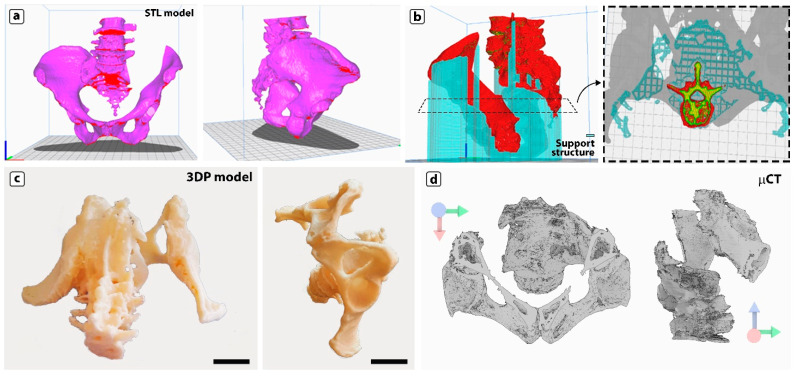
(**a**) 3D CAD model of the iliac crest with overhanging surfaces evidenced in red. (**b**) Supporting structures (shown in blue) for the overhanging parts from a lateral view and from the top show the square grid pattern. (**c**) Photographs of the printed model from two different views. (**d**) Computed X-ray microtomography (μCT) 3D rendering of the printed model from frontal and lateral views.

In numerous biomedical fields, 3D printing plays a key role, particularly in fabricating customised prostheses and temporary scaffolds for tissue engineering. Creating 3D models of tissue abnormalities or carcinomas is an underappreciated medical application that aids surgeons in precise preoperative planning and evaluating several clinical, anatomical, and prosthetic aspects to optimise results and prevent complications [52,53]. In this picture, we tried to reproduce, at a 1:2 scale, the sacroiliac joint of a patient affected by chondrosarcoma. Figure 10a reports the CAD model elaborated from computed tomography (CT) scans. Due to the anatomical intricacy and existence of many concave surfaces, careful setting of the printing parameters, especially the supporting structure, was necessary (Figure 10b). The supporting structure had to be designed to prevent the vertebra from becoming embedded, making its removal easier without damaging the surface of the anatomical model. As shown in Figure 10b, the concentric layout of the supporting structure is ideal for preserving the quality of the printed model since it has fewer contact points with the underlying anatomical model, hence minimising the potential damage caused by its removal. The optimal printing settings are summarised in Tables S2 and S3.

Figure 10c provides a few representative images of the model from various angles, and the 3D μCT rendering (Figure 10d) enables appreciation of the model’s high accuracy level from the external and internal views. These results demonstrate the PHBV–PLA50:50 blend suitability as material for FDM.

## 5. Conclusions

The topic of sustainability and circular economy has also impacted the world of 3D-printing materials concerning FDM. The printability of commercially available PHBV–PLA blends with two distinct weight ratios was tested in this study. We demonstrated that printability depended on the melt flow properties and the development of a crystalline phase upon cooling. The rheological behaviour at low shear rate and frequency and the thermal characterisation put into evidence the biphasic nature of the blend both in the molten and solid state, thereby corroborating earlier research findings. The study of melt rheology allowed for correlating the viscoelastic properties with the first normal stress exerted on extruded melt filament, which, theoretically, should determine the extent of die-swelling. Surprisingly, an inverse correlation was found between the first normal stress and filament diameter. Cole–Cole plots provided valuable insights showing that the relaxation time is the critical parameter governing the degree of die-swelling. The longer relaxation time observed for PHBV/PLA25:75 indicates that the molten material has a more significant opportunity to die-swell before solidification leading to larger fibres. As a result, at the same extrusion speed and nozzle diameter, PHBV/PLA50:50 enables the deposition of thinner fibres and achieves a better resolution in comparison to PHBV/PLA25:75. Another consequence of the relaxation time is on the %flow. PHBV/PLA25:75 has a longer relaxation time and produces larger fibre diameters at all extrusion speeds. Therefore, to counteract die-swelling, the %flow must be adjusted to lower values than PHBV/PLA50:50, slowing down the printing process.

A critical issue that emerged during printing tests, especially large objects, is the distortion of printed objects. Warping is caused by the non-uniform shrinkage of the polymer during the transition from a molten state to a solid state. In the present case, warping is driven by the crystallisation of the PHBV phase. The blend richer in PHBV content underwent a higher degree of warping. The temperature of the build plate turned out to be crucial in minimising warping. Keeping the deposition plate temperature at the PLA’s *T_g_* helped reduce the differential shrinking and prevented warping. In this way, warping was suppressed entirely in PHBV/PLA50:50 and significantly reduced in the case of PHBV/PLA25:75.

Combining all the experimental data and the optimal printing conditions drawn from them indicates that the PHBV–PLA50:50 presented an excellent printability potential. This proved true, as demonstrated when creating a technical item and replicating geometrically complex anatomical parts with good shape fidelity and integrity. Taken as a whole, the obtained outcomes also pinpoint the future interest in testing blends made of different PHAs (both in terms of higher HV content and in terms of different monomeric compositions), to enlarge the application of these materials in the FDM field.

## Figures and Tables

**Table 1:** Sample thermal properties: glass transition temperature (*T_g_*), specific heat variation at *T_g_ (*∆*C_p_*), enthalpy of melting (∆*H_m_*) and of cold crystallisation (∆*H_cc_*), the temperature of cold crystallisation (*T_cc_*) and of melting (*T_m_*) of PLA, PLA/PHBV50:50 and PLA/PHBV25:75.

	1st Heating						1st Cooling				2nd Heating					
Sample	*T_g_* (°C)	∆*C_p_* (J g^−1^ K^−1^)	*T_cc_* (°C)	∆*H_cc_* (J g^−1^)	*T_m_* (°C)	∆*H_m_* (J g^−1^)	*T_g_* (°C)	∆*C_p_* (J g^−1^ K^−1^)	*T_mc_* (°C)	∆*H_mc_* (J g^−1^)	*T_g_* (°C)	∆*C_p_* (J g^−1^ K^−1^)	*T_cc_* (°C)	∆*H_cc_* (J g^−1^)	*T_m_* (°C)	∆***H_m_* (J g^−1^)**
PLA	60	0.51	119	21	152	24	54	0.5	-	-	58	0.50	119	22	150	23
PLA/PHBV50:50	53	0.27	116	5.41	155–176	55	54	0.276	121	46	2 ^a^ 57	- 0.27	126	4	138–174	53 ^b^
PLA/PHBV25:75	52	0.15	-	-	177	64	53	0.154	120	62	5 ^a^ 57	- 0.12	114	5	154–175	76 ^b^

^a^ ∆*C_p_* not evaluable. ^b^ Total melting enthalpy calculated from the two peaks.

**Table 2:** Zero-shear viscosities (*η*_0_) and relaxation times (*τ*), determined through the Cole–Cole plot, for the two blends determined at two different temperatures.

	*η*_0_ (Pas × s)		*τ* (s)	
	180 °C	190 °C	180 °C	190 °C
PLA/PHBV50:50	8870	4820	0.10	0.05
PLA/PHBV25:75	3050	2000	0.04	0.02

**Table 3:** Young moduli (*E*), yield point (*σ_yield_*), yield strain (*ε_yield_*) of cylindric specimens under compression.

Cylinders	*E* (GPa)	*σ_yield_* (Mpa)	*ε_yield_* (%)
PLA/PHBV100:0	1.3 ± 0.15	73 ± 3	8
PLA/PHBV50:50	1.23 ± 0.03	72 ± 2	8
PLA/PHBV25:75	0.88 ± 0.02	61 ± 2	8

**Table 4:** Young moduli (*E*), yield point (*σ_yield_*), yield strain (*ε_yield_*) of cubic specimens under compression in a direction parallel (//) or perpendicular (⊥) to the printing direction.

	Compression // to Z			Compression ⊥ to XY		
Cubes	*E* (GPa)	*σ_yield_* (Mpa)	*ε_yield_* (%)	*E* (GPa)	*σ_yield_* (Mpa)	*ε_yield_* (%)
PLA/PHBV100:0	1.23 ± 0.03	77 ± 6	8	1.22 ± 0.01	72 ± 5	8
PLA/PHBV50:50	1.14 ± 0.01	70 ± 2	9	1.05 ± 0.03	61 ± 12	9
PLA/PHBV25:75	0.74 ± 0.02	62 ± 2	20	0.86 ± 0.02	60 ± 14	13

**Table 5:** Young moduli (*E*), tensile stress (*σ_break_*), deformation at break (*ε_break_*) of PLA and PHBV blend of different weight ratios printed into dumbbell specimens deposited according to different raster angles.

	Raster Angle = 0°			Raster Angle = 90°		
Specimens	*E* (GPa)	*σ_break_* (Mpa)	*ε_break_* (%)	*E* (GPa)	*σ_break_* (Mpa)	*ε_break_* (%)
PLA/PHBV100:0	0.16 ± 0.01	12 ± 1	9	0.19 ± 0.003	8.5 ± 0.7	6
PLA/PHBV50:50	0.15 ± 0.01	12 ± 1	13	0.17 ± 0,003	2.5 ± 0.7	2
PLA/PHBV25:75	0.11 ± 0.01	0.9 ± 1.2	2	0.14 ± 0.001	8.3 ± 0.2	6

## Data Availability

The original contributions presented in the study are included in the article/Supplementary Materials, further inquiries can be directed to the corresponding authors.

## Supplementary Materials

The following supporting information can be downloaded at: https://www.mdpi.com/article/10.3390/jfb16010009/s1, Figure S1. Definition of warping degree. Schematics of a specimen deformed by warping viewed frontally. *d* and *h* are the measurable parameters used for the calculation of the warping degree according to the formula reported in the experimental section. Figure S2. (a) schematics of specimen printed at different extrusion speeds: in (b), the range covered is up to 50 mm/sec, and using nozzles with a diameter of 0.4, 0.5, 0,6 mm; in (c) the range explored is between 70 – 120 mm/sec, nozzles used have a diameter of 0.5 and 0.6 mm. In (d) the specimen was fabricated setting the extrusion speed at 70 mm/min, in (c) 120 mm/min. Nozzle diameter = 0.6 mm. Figure S3. (a) CAD model of dumbbell with a brim layer; (b) warping angle as a function of first layer % flow with and without brim. The blend used was PLA/PHBV25:75. Figure S4. Influence of printing speed (*v*) on the warping angle of parallelepiped specimens obtained from the two blends: PLA/PHBV50:50, PLA/PHBV25:75. Figure S5. Stress vs strain curves under compression of printed cubic ((a), (b)) obtained using PHBV/PLA50:50, PHBV/PLA25:75 blends and PLA by applying the stress in a direction (a) perpendicular and (b) parallel to the deposition plane. In (c) the stress/strain curve for the cylindrical is reported. Figure S6. Stress vs strain curves under traction of printed dog-bone specimens obtained using PHBV/PLA50:50, PHBV/PLA25:75 blends and PLA. Raster angle (a) 0°, (b) 90°C. Table S1. Optimised printing parameters of the shaft support. Table S2. Optimised printing parameters of the iliac crest. Table S3. Optimised printing parameters for the support of the iliac crest.
