# Supplementary material for: Study of the Interplay Among Melt Morphology, Rheology and 3D Printability of Poly(Lactic Acid)/Poly(3-Hydroxybutyrate-Co-3-Hydroxyvalerate) Blends
Source: J Funct Biomater. 2025 Jan 1;16(1):9. doi: 10.3390/jfb16010009 (PMC11766440; doi:10.3390/jfb16010009)
Supplement: Supplementary file 1 [file jfb-16-00009-s001.zip › jfb-3378611-supplementary.pdf]

SUPPORTING INFORMATION

Study of the Interplay Among Melt Morphology, Rheology and 3D Printability of Poly(Lactic Acid)/Poly (3-Hydroxybutyrate-Co-3-Hydroxyvalerate) Blends

Marco Costantini [1,*], Flavio Cognini [2], Roberta Angelini [3,4], Sara Alfano [5], Marianna Villano [5], Andrea Martinelli [5], David Bolzonella [6], Marco Rossi [2] and Andrea Barbetta [5,*]

[1] Institute of Physical Chemistry, Polish Academy of Sciences, 01-224 Warsaw, Poland
[2] Department of Basic and Applied Sciences for Engineering, Sapienza University of Rome, 00161 Rome, Italy; flavio.cognigni@uniroma1.it (F.C.); marco.rossi@uniroma1.it (M.R.)
[3] Institute for Complex Systems, National Research Council (ISC-CNR), Sapienza University of Rome, P.le A. Moro 2, 00185 Rome, Italy; roberta.angelini@roma1.infn.it
[4] Physics Department, Sapienza University, P.le Aldo Moro 2, 00185 Rome, Italy
[5] Department of Chemistry, Sapienza University of Rome, P.le Aldo Moro 5, 00185 Rome, Italy; sara.alfano@uniroma1.it (S.A.); marianna.villano@uniroma1.it (M.V.); andrea.martinelli@uniroma1.it (A.M.)
[6] Department of Biotechnology, University of Verona, Via Strada Le Grazie 15, 37134 Verona, Italy; david.bolzonella@univr.it
* Correspondence: mcostantini@ichf.edu.pl (M.C.); andrea.barbetta@uniroma1.it (A.B.)

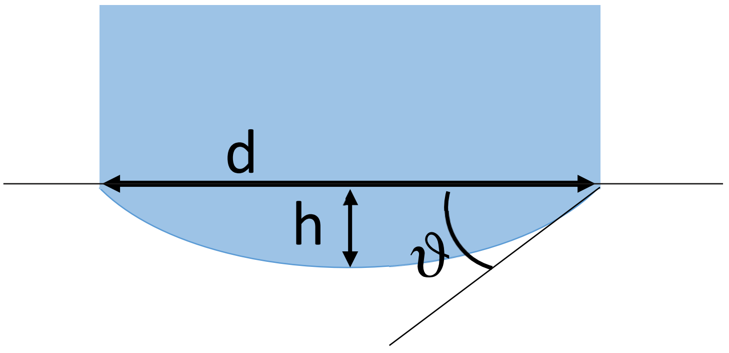

Figure S1. Definition of warping degree. Schematics of a specimen deformed by warping viewed frontally. *d* and *h* are the measurable parameters used for the calculation of the warping degree according to the formula reported in the experimental section.

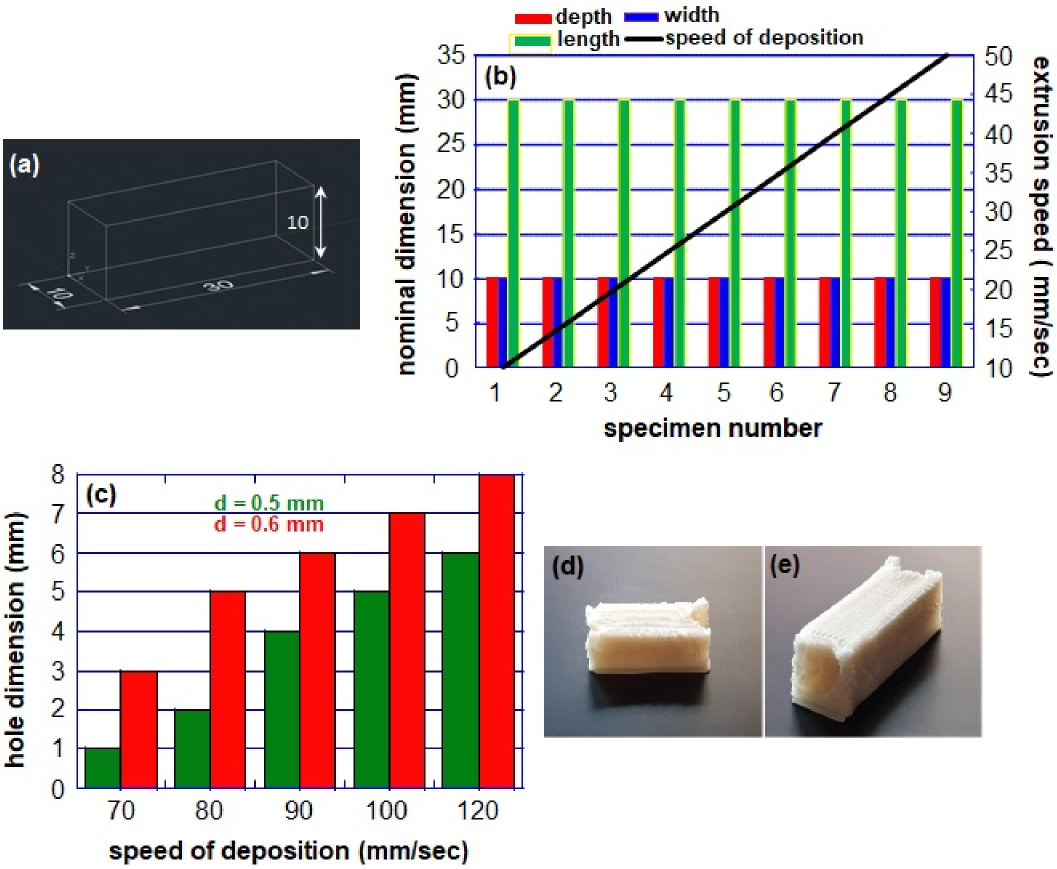

Figure S2. (a) schematics of specimen printed at different extrusion speeds: in (b), the range covered is up to 50 mm/sec, and using nozzles with a diameter of 0.4, 0.5, 0,6 mm; in (c) the range explored is between 70 – 120 mm/sec, nozzles used have a diameter of 0.5 and 0.6 mm. In (d) the specimen was fabricated setting the extrusion speed at 70 mm/min, in (c) 120 mm/min. Nozzle diameter = 0.6 mm.

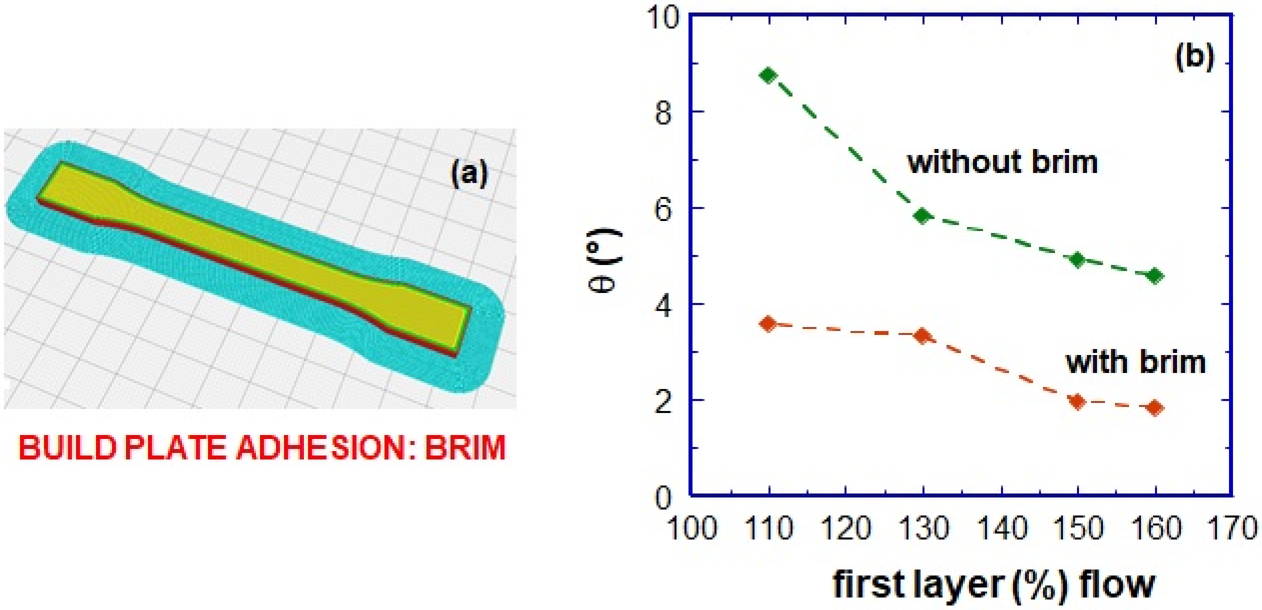

Figure S3. (a) CAD model of dumbbell with a brim layer; (b) warping angle as a function of first layer % flow with and without brim. The blend used was PLA/PHBV25:75.

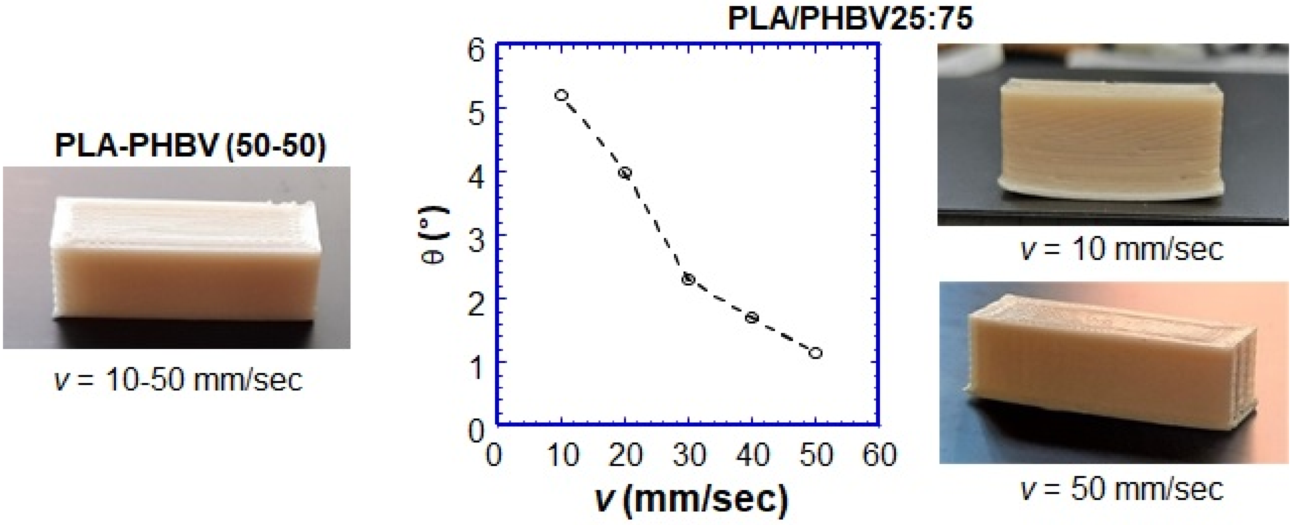

Figure S4. Influence of printing speed (v) on the warping angle of parallelepiped specimens obtained from the two blends: PLA/PHBV50:50, PLA/PHBV25:75.

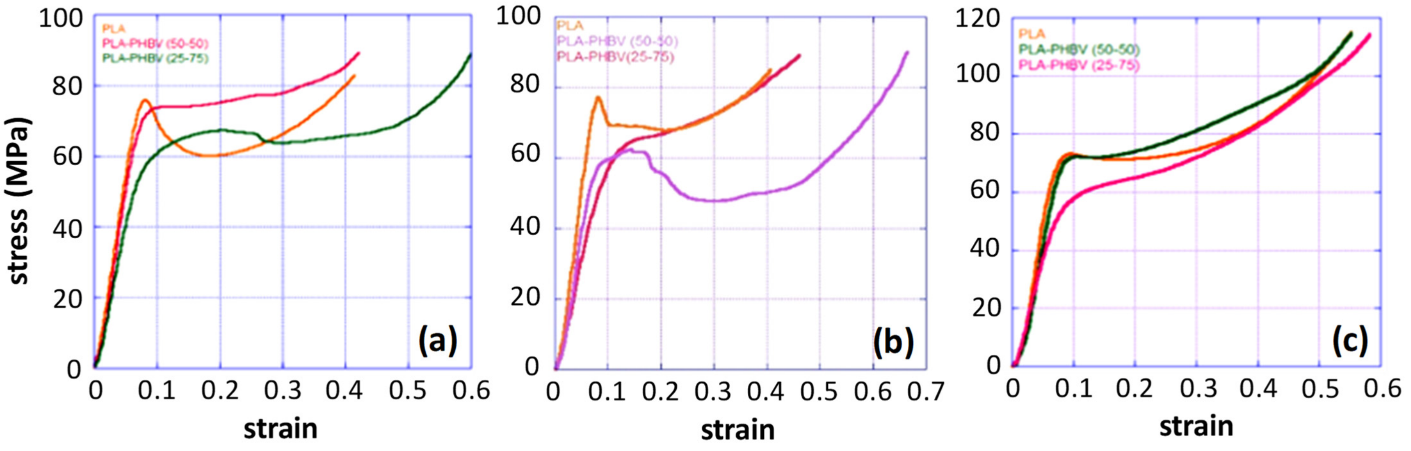

Figure S5. Stress vs strain curves under compression of printed cubic ((a), (b)) obtained using PHBV/PLA50:50, PHBV/PLA25:75 blends and PLA by applying the stress in a direction (a) perpendicular and (b) parallel to the deposition plane. In (c) the stress/strain curve for the cylindrical is reported.

PLA
PLA/PHBV50:50
PLA/PHBV25:75

PLA
PLA/PHBV50:50
PLA/PHBV25:75

PLA
PLA/PHBV50:50
PLA/PHBV25:75

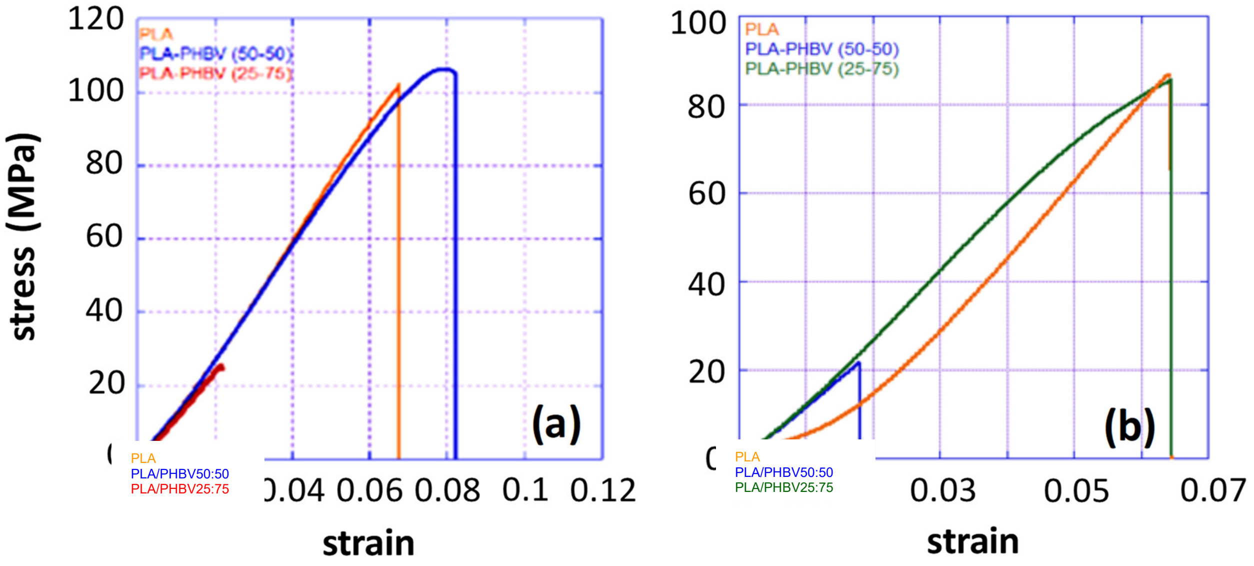

Figure S6. Stress vs strain curves under traction of printed dog-bone specimens obtained using PHBV/PLA50:50, PHBV/PLA25:75 blends and PLA. Raster angle (a) 0°, (b) 90°C.

Table S1. Optimised printing parameters of the shaft support.

Printing parameters		Units
Layer height	0,1	mm
Line width	0,4	mm
Wall thickness	1,2	mm
Top/bottom thickness	1,2	mm
Pattern	Line	
Pattern line direction	90	°
Infill density	10	%
Infill pattern	Grid	
Infill pattern line direction	45	°
Printing temperature	190	°C
Build plate temperature	50	°C
Flow	95	%
Innitial layer flow	130	%
Printing speed	50	mm/sec
Infill printing speed	40	mm/sec
Initial layer printing speed	40	mm/sec
Retraction distance	0,3	mm
Fan cooling	20	%
**Support type**	**Normal**	
Support placement	Touching build plate	
Support pattern	Lines	mm
Support line distance	1	mm
Support Z distance	0,15	mm
Support x/y distance	0,4	mm
Build plate adhesion	raft	
Raft extra margin	30	mm
Raft air gap	0,15	mm
Raft top layers	5	
Top layer thickness	0,1	mm
Top line width	0,4	mm

Table S2. Optimised printing parameters of the iliac crest.

Printing parameters		Units
Layer height	0,1	mm
Line width	0,4	
Wall thickness	1,2	
Optimize wall printing order	Activated	
Compensate wall overlaps	Activated	
Top/bottom thickness	1,2	
Top/bottom pattern	lines	
Infill density	0	%
Printing temperature	180	°C
Build plate temperature	50	°C
Flow	95	%
Initial layer flow	150	%
Print speed	50	mm/sec
Retraction distance	0,3	
Fan speed	20	%

Table S3. Optimised printing parameters for the support of the iliac crest.

Number of tests	Support structure	
**Test 1**	Normal support	
	Support placement	Touching build plate
	Support pattern	grid
	Support wall line count	1
	Support density	20 %
	Support z distance	0,3 mm
	Support x/y distance	3 mm
**Test 2**	Normal support	
	Support placement	Touching build plate
	Support pattern	concentric
	Support wall line count	1
	Support density	13 %
	Support z distance	0,4
	Support x/y distance	4
